# Change and Continuity in Vaping and Smoking by Young People: A Qualitative Case Study of A Friendship Group

**DOI:** 10.3390/ijerph15051008

**Published:** 2018-05-17

**Authors:** Neil McKeganey, Marina Barnard

**Affiliations:** Centre for Substance Use Research, Block 3/2 West of Scotland Science Park, Kelvin Campus, Maryhill Road, Glasgow G20 0SP, UK; barnard@csures.org

**Keywords:** smoking vaping, young people, transition

## Abstract

This paper reports a qualitative case study of a small friendship group (*n* = 8) in Glasgow, Scotland. Interviewed twice at six months apart, these 16 to 17 year olds reported a substantial change in their use of and attitudes towards e-cigarettes and tobacco. At time 1, vaping generated much excitement and interest, with six out of eight individuals having their own vape device. At time 2, only two young people still vaped, with the others no longer professing any interest in continued vaping. The two regular smokers, who had been smoking before they first vaped, now only vaped privately and to reduce their tobacco intake. This small case study illustrates plasticity in the use of these devices; just as young people can move into their use, so too can they move away from them. This small study underscores the importance of differentiating between long-term, frequent, consistent use and more episodic, experimental and infrequent use by young people and for undertaking a measurement of actual e-cigarette use at multiple time points in both quantitative and qualitative studies. In addition, the case study illustrates the powerful impact which peers can have on teenagers use of e-cigarettes.

## 1. Introduction

The introduction, proliferation, and diversification of e-cigarettes in the tobacco, nicotine, and smoking marketplace has generated concern that these devices may act as a gateway to smoking and a way of re-normalising smoking [1,2,3]. Those concerns have been fuelled in part by research indicating a marked increase in the use of electronic nicotine delivery systems (ENDS) by young people, and by the findings from a range of surveys that have suggested that young people who have used e-cigarettes are more likely than their non e-cigarette using peers to have started smoking when followed-up over a twelve-month period [4,5]. Within the United States, Soneji and colleagues undertook a systematic review and meta-analysis of nine longitudinal studies exploring the relationship between adolescent use of e-cigarettes and the subsequent likelihood that the young people involved will go on to use combustible tobacco products [6]. According to these researchers, “e-cigarette use is associated with an increased risk of future cigarette smoking initiation and current cigarette smoking even after adjusting for potentially confounding demographic, psychosocial, and behavioural risk factors”. Within the UK, by contrast, researchers have not found evidence of such a strong relationship between adolescent e-cigarette use and the subsequent initiation of smoking. Bauld et al. [7] drew upon data from five large UK surveys of young people aged 11 to 16. Whilst these studies demonstrated that between a tenth and a fifth of 11–16 year olds had tried e-cigarettes, only around 3% were using e-cigarettes on a weekly basis, 99.5% of whom were smoking at the point at which they initiated e-cigarette use. As these researchers point out, “Our findings indicate that there is no evidence of e-cigarettes driving smoking prevalence upwards. This is important and suggests that fears about e-cigarettes as a gateway to more youth becoming smokers are not currently justified, at least in the UK” [7].

Alongside attempts at quantifying the possible impact of early e-cigarette use on the likelihood of adolescents going on to smoke combustible tobacco products, attention has also focussed more recently on measuring the extent of transitions in adolescents’ e-cigarette and smoking behaviour. Hair et al. [8] analysed data from a nationally representative longitudinal survey of 15 to 21 year olds in the U.S. (*n* = 15,275). These researchers were able to show substantial movement into and out of different patterns of use, for example, those young people who only used combustible tobacco products, or who were dual users of vaping and combustible tobacco products, tended to do so for around six months before changing to a different user state, whilst those who used vapor products tended to do so for three months before transitioning to a different user state. This suggests that at least amongst young people, the use of e-cigarettes is a good deal more fluid than might have initially been thought. In the light of that, it is perhaps understandable that surveys in both the UK and U.S. have found a marked reduction in proportions of young people reporting having used e-cigarettes on an “ever basis” (i.e., at any time in the past) and those reporting using e-cigarettes within the last 30 days or on at least 20 of the last 30 days [9,10].

Whilst the use of e-cigarettes has often been framed in terms of these devices acting as a means to consume nicotine, in fact, qualitative research into the use of e-cigarettes by young people has tended to stress the issues of pleasure and social playfulness rather than nicotine. Pokhrel and colleagues [11] reported that the young adult vapers (aged 18–35) they interviewed explained the appeal of vaping in terms of the activity being novel and “cool” and as forming part of their socialising with friends. Similarly, Measham and colleagues [12] found that young people’s motivations for using e-cigarettes tended to focus on issues of pleasure and fun rather than on the need to meet their addiction to nicotine. Alongside these qualitative studies, Miech and colleagues [9] have drawn upon large scale survey data to show that adolescents in the U.S. are more likely to be vaping non nicotine containing flavoured e-liquids than e-liquids containing nicotine.

As concern has grown over the extent of e-cigarette use by adolescents, there has been an increase in attention focussed on the measures used to establish the frequency of e-cigarette use. Criticism has been directed, for example, at the reliance in some studies on “any past use” of e-cigarettes or measures of the recency of e-cigarette use which do not necessarily accurately convey the frequency of current use [13]. Villanti and colleagues [14] have stressed the importance of precise measurement in assessing the public health impact of e-cigarettes. Warner [15] has similarly emphasised the importance of being able to distinguish between experimental, infrequent use of e-cigarettes by young people and that which is more frequent and more long-term. In-depth qualitative research can usefully complement prevalence data by providing a detailed picture of contextual factors influencing the increased use of these devices by young people, their place within youth cultures, and their significance with respect to tobacco smoking. In this paper, we report data from a research study that was designed to explore the possible fluidity in young peoples’ perceptions and engagement with e-cigarettes by re-interviewing the members of a small teenage friendship group in Glasgow, Scotland, focussing on how their relationship and perception of e-cigarettes changed over a six-month period.

## 2. Methods

The eight young people who we focus upon in this paper were interviewed as part of a larger study in which we focussed on the perception and engagement with e-cigarettes amongst 100 non smoking vapers aged between 16 to 30 and 50 vapers [16,17]. The aim of this wider study was to explore attitudes towards vaping and smoking and to consider whether the availability and public visibility of e-cigarettes had resulted in smoking becoming more socially acceptable within Scotland. Predominantly, the interviewees in our larger study viewed vaping as a means to reduce the harms of smoking with very few describing vaping as a source of fun and enjoyment and as something that might appeal to both smokers and non-smokers. In contrast, the eight young people who formed the basis of this case study were much more inclined, at least initially, to describe vaping as a fun activity that was appealing to both smokers and non-smokers and which formed part of their social and leisure activities with friends.

The eight respondents who formed this case study were interviewed on two occasions, six months apart. Interviews focussed on topics such as the young person’s perception and use of e-cigarettes, the circumstances of their use, the reactions of others to their use, the reasons for their use, the frequency of their use of these devices, and their likelihood of using e-cigarettes in the future. The eight participants who formed this case study attended the same fee paying (i.e., private) school, spending time with each other socially as well as during school time. In Table 1 below we have summarised the smoking and vaping behaviour of the young people we were interviewing. At the point of their first interview (T1), all of our interviewees were aged 16. By the time of their second interview (T2) six months after the first interview, three had turned 17 years old. Most of the original peer group were male (6/8). At T1, two males reported that they were current smokers; additionally, one male and one female reported that they smoked tobacco at parties. By T2, one other male had become a smoker and another four said that they smoked occasionally at parties. Vaping at least once a week was reported by six out of eight individuals at the time of their first interview. However, by the time of their second interview, only two of the group were vaping; both of whom reported that they were current smokers and that they were vaping on a daily basis.

## 3. Results

At the time of the first set of interviews, the subject of e-cigarettes appeared to elicit a good deal of interest and excitement, even by the two participants who reported that they had not vaped beyond trying it out once. As has been reported elsewhere, e-cigarettes can generate a lively interest among young people [12]. Amongst the friends interviewed here, vaping was commonplace, all of them had tried it, and six of them had purchased at least one vape device (not e-cigarettes) in the recent past. The sight of young people vaping was regarded as something of an “event”, a “performance” or “show” that rapidly drew the attention of others in their wider social group and afforded them a certain social status:

…and we bring it to parties and stuff and people are like “oh that’s so cool” … you can do tricks and stuff and maybe because it’s a new thing as well it surprises them with the massive like clouds.

(Daniel: smoker, T1)

During the first set of interviews, there was a palpable sense of “play” and sociality associated with vaping, which appeared to have influenced the decision to purchase a device for some:

It was my birthday and I just decided to buy one. I had leftover cash and everyone was doing it. Maybe that’s not the way I should be doing things because everyone else is but I thought that kind of looks like an enjoyable pastime so I thought I’ll go for it and bought a cheap one.

(Josh: non-smoker, T1)

It was difficult to definitively characterize the quantity of smoking and vaping these young people engaged in because it appeared to be highly context dependent. Although two out of eight interviewees described themselves as regular, daily smokers who also regularly vaped, there were varying degrees to which the others vaped or smoked. In total, four young people said they vaped at least once a week and used a nicotine infusion of 0.3 mg. Two young people who owned vape devices said they vaped “sometimes”, mostly when in the company of their friends and if they were “bored”. The remaining two said they had only ever tried it out of curiosity and were not intending on doing it more than this. Of the six who had purchased at least one vape device, two said that they used 0mg nicotine and vaped only intermittently. The two young people who did not own vape devices said that whilst they often enjoyed watching their friends vaping and the tricks they could perform, they were not personally interested in vaping:

I’ve never done it properly. I just don’t have an interest in it. I didn’t really like it that much. It was kind of just really weird. I didn’t really like it. I like seeing the way it moves but I don’t really do it.

(Gayle T1)

Four of the young males interviewed described themselves as vaping regularly, with three of them saying they vaped multiple times every day. The fourth young man said he used it on alternate nights because he was “bored and it’s there”. The situational character of much of this vaping can be heard in the following interview excerpt:

If it’s people I don’t really know who are doing it and they’re like “do you want a draw?” then no. But if it’s like my best friend then I can just pick it up and do it when I’m bored. I’ve never had a desire to do it, it’s just if it’s there and I can do cool things with it. Just something to play with. It’s not like I need to try it, like I feel I need to vape ... I just like blowing circles with the smoke and trying like tricks and stuff just like that, it’s kind of interesting seeing how smoke works.

(Lyra: non-smoker, T1)

Even in this small group, there were very different styles of use and it was clear that shifting social circumstances influenced the likelihood of use:

I have phases where I’ll go through a lot of the e-juice at the one time and then I’ll just go off it for a wee bit and then just have the occasional one at night … When I was studying over the prelims I went through so many bottles because I hate studying and it was just something to do.

(Daniel T1)

Social gatherings, particularly parties, were occasions where vaping was likely to be seen, experimented with, and sometimes resulted in the initiation of use [18].

I saw one at a party and thought it was cool so I decided to get one to take to a party.

(Martyn T1)

These narratives gave the clear impression of a group of young people for whom vaping was a pleasurable, social activity, whether in their use of the device, the flavours and the plumes of smoke they could generate, or in the kudos of their skilled display of tricks to their peers. Their motivations for using e-cigarettes or vapes were not, however, framed in terms of the infusion of nicotine in the liquids and they did not appear to see these devices as cigarette substitutes.

### 3.1. Smoking and Vaping Hierarchies

These young people described a perceived hierarchy between smoking and vaping. Whilst vaping was perceived as being “cool”, it was not seen as being as “cool” as smoking:

People still say smoking is cooler than vaping because see, like at a party you’ll see people smoking still, they’ll go outside to have a smoke, a social smoke, nobody would go outside to have a social vape.

(Gavin T1)

When asked how likely it was that they would continue to vape a year into the future, there was a decided view that this behaviour was likely to be a phase rather than a fixed pattern of behaviour. The three who appeared to be the most committed to vaping had seen a drop off in interest among their peer group:

We’re the only people that have kind of kept it on, a lot of our friends will maybe toke it once every two, three weeks … We’ve got about 10, 15 friends who do it but they don’t do it on a regular basis like us, they’ll keep it in a drawer.

(Thomas T1)

They themselves could envisage a time when their interest in vaping would taper off. The widely held perception of cigarettes as the “real thing” relative to e-cigarettes might possibly have influenced their view of vaping as a phase to grow out of:

Sometimes smokers look down on vapers, they’re like “are you not cool enough for that”? Clive’s brother he made fun of me because he was like “Och are you not hard enough to do the real thing”, he went on “you’re too scared to do the real thing”. … Sometimes at parties’ people will say “that’s so gay, what are you doing with that why don’t you have a real cigarette”?

(Martyn T1)

The two young people who regularly smoked felt that their vaping was healthier than smoking tobacco and helped reduce their dependence on cigarettes:

A lot of people say they don’t know if it’s healthier or not (vaping), but when I smoke because I play rugby a lot I can feel it in my lungs, whereas when I go for a vape my lungs feel fine. Vaping has changed the way I see smoking. I feel bad when I have a cigarette now … I feel unhealthy. I feel I’m putting chemicals and cancer and stuff in my body, whereas like before I didn’t really care.

(Thomas T1)

The six vapers personally experienced vaping as being better for them than smoking (“my chest is not tight”) and thought that as vaping had been invented to help people stop smoking, it must be less harmful, particularly when they were vaping zero nicotine. None of the young people, however, thought vaping was without harm:

I know they were created to stop people from smoking … I would say there is still some harm in them because it is still chemicals going into your body and people do smoke the nicotine ones … but it’s not as bad as smoking.

(Lyra T1)

Only one young man described his vaping as addictive. Whilst viewing it as a safer alternative to smoking, the issue of nicotine dependence had surfaced such that if he could not vape he said he would almost certainly smoke more. The other three regular vapers did not consider this to be their situation. They discounted addiction for two reasons: firstly, they did not consider that they “needed” to vape and instanced having spent weeks away from vaping without adverse consequences or even particularly noticing. Secondly, they used very low concentrations of nicotine or none at all. This pattern of low or, in most cases, zero nicotine, was replicated across all six of the young people who had been sufficiently interested in vaping to invest in a device at T1.

### 3.2. Change and Continuity: Six Months Later 

At T2, six months later, we successfully re-interviewed seven of the eight young people. Even in this relatively short time period, there had been some notable shift in young peoples’ perception of e-cigarettes.

By the T2 interviews, attitudes towards vaping were much less positive, the excitement and novelty of e-cigarettes was no longer mentioned, and e-cigarettes were no longer apparently a part of their socialising with friends. At T1, most (6/8) owned a vape device and said they vaped more than once a week. Six months later, only two (2/7) had functioning devices that they used at least once a week. The two young people who continued to vape were also regular smokers, one of who had moved from intermittent to regular smoking in the time between the interviews.

The young people who said they no longer vaped were also those who did not consider themselves to be smokers. Problems with devices breaking and the expense of buying liquids and maintaining vaping equipment had often precipitated the decision to stop a behaviour that they no longer felt invested in:

It kept breaking and I couldn’t be arsed with it. I didn’t need to get off smoking or anything so it was just like pointless, there wasn’t any need to do it and I didn’t like it much, it makes me feel lightheaded, made me feel sick.

(Lyra T2)

The rationale for Lyra’s vaping between these two time points altered from describing vaping as something she did when she was “bored” or “to do cool things with it” at T1, to being “pointless” since she was “neither a smoker nor nicotine dependent” at T2. Similarly, the enthusiasm for vaping that had earlier led one young man to use his birthday money to buy a vape had steeply declined six months on:

I did have a vape but I didn’t use that much. It was just a thing that was there, that I would just sort of pick up whenever I felt like it and now I’m much the same but I don’t use the vape at all and I’m actually trying to sell it … the vape has just become a drag. It’s like one of those things you need to do and it’s a lot of money and I don’t really care about it all that much to keep it up like a hobby so I’m just going to sell it. … I quite enjoyed it in the first few weeks I had it but I kind of got bored.

(Josh T2)

The price of e-liquids, and the effort and cost of vape device maintenance were factors that were cited as inhibiting regular or continued vaping:

I vaped quite a lot in July and I still vape but I can’t really afford the liquid so I don’t buy it that often because they’re constantly putting the prices up so I don’t buy it … I stopped when I couldn’t afford it anymore, midway through my exams I just wasn’t getting any more so there was no point, I was just wasting it all on that.

(Gavin T2)

The two dual vaper/smokers noted that the relatively high, upfront, costs of vaping sometimes resulted in their buying cigarettes as a cheaper alternative:

Daniel: Because I always end up like breaking it or something. I’d say with a vape you’ve got to buy juice, it’s like you buy it in bulk so you need lots of money to buy it … so say if I don’t have a tenner on me…

Thomas: It’s cheaper to go buy tobacco (Daniel and Thomas T2)

Further, these two young people considered that cigarettes were “more reliable” than vape devices because nothing went wrong with them. They were more convenient to use and also less likely to be stolen, especially at parties. Such rationales led to a preference to smoke tobacco over vaping in those situations.

### 3.3. Vaping and Smoking

A perceived public disapproval of vaping and perhaps more importantly, a negative orientation towards vaping amongst their peers generally, exerted an important influence. In just six months, vaping had gone from being a fun, cool thing to do at parties to being somewhat passé. To avoid drawing negative comments from other people around them, the two regular vapers reported vaping in private and cigarette smoking in public, particularly at parties. An awareness of a public aversion to vaping had surfaced in the interviews six months earlier but was more prominent at T2:

I think a lot of people have been scared off because see when you’re walking down the street like blowing a cloud of whatever people of every age, they’re just like … the looks you’ll get from people. So, I think that’s why you don’t see many on the street nowadays.

(Thomas T2)

Perhaps more pertinently, the negative comment attached to vaping at parties was allied with an explicit ranking of smoking cigarettes over vaping:

…people just kind of mess with you a bit when you’ve got it, people can undermine you a bit and say, “Ha, what a gimp he’s vaping, you know”.

(Daniel T2)

Smoking at parties was reported by five out of seven young people at T2. Social smoking appeared to mean sharing puffs of others’ cigarettes, or, having one or two cigarettes with friends. It was seen by these young people as a limited, situational activity.

When first interviewed, Daniel reported that he occasionally smoked and regularly vaped using 0mg nicotine liquids. Six months later, he was a regular smoker and also vaped a higher nicotine content between:

I started getting nicotine in the juice because my friends were and I thought you know it’s quite cool getting some nicotine in, see what it’s like. At first, it’s quite harsh on your throat, but then you get used to it, and then, you kind of stop thinking about it. Like it’s kind of subconscious- 0.3 mg a weak bit of nicotine in it and also you vape it more when you’ve got nicotine.

(Daniel T2)

The higher nicotine content was allied to the particular vape device he now used, but for the other teenager, it was clearly associated with wanting the nicotine:

…over the summer, I smoked a lot so I found I couldn’t go back to the 0.3 mg, but then I can have 3 but sometimes say like 5 is good because you don’t have as much time, 3 is ok if you’re constantly doing it whereas at school you don’t have time to do that, v so doing it at lunchtime or break time 6 is better, a couple of tokes.

(Thomas T2)

Daniel described how over the last few months a pattern of dual use of both smoking and vaping had occurred:

I go through a phase like I’ll vape for a month and then like smoke for a week and then by the end of the week I’m done with smoking so I’ll go vape for another while…

(Thomas T2)

Daniel described his own combined vaping and smoking although he stated that he preferred vaping to smoking because he enjoyed the flavours and the actual experience of vaping, and wanted to limit his exposure to the dangers of smoking:

My friend is always giving me cigarettes he’s like “c’mon I’ll give you one” because he wants to smoke with someone, and then I’ll do it and I’ll be like “no” and I’ll throw it away. Then I’ll pick my vape up again but if I’ve not been vaping for a while, like a whole day, I’ll have a cigarette at the end of the day. It’ll be quite nice because I’ve not had any nicotine in my system for the whole day.

(Daniel T2)

These comments indicate the contingent nature of smoking and vaping, at least for these young people. Involvement in both of these activities was dependent upon who they were with, the situations they were in, the reaction of others, and their general feelings towards vaping and smoking. These two young people saw vaping as a brake on smoking and also as a relaxing, but now private, pleasure in its own right. In the six months between interviews, their attitudes towards vaping and indeed smoking had changed quite significantly. Their reported engagement in vaping and smoking suggested patterns of behaviour that were fluid and variable rather than entrenched and fixed.

## 4. Discussion 

In recognising the limitations of our research, it is important to note that our study is based entirely on self-report with no independent confirmation of the young peoples’ reports of their vaping or smoking. Similarly, with such a small sample, it is not possible to know how representative our interviewees were of other young people. As a result, we cannot comment on the likely proportion of young people adopting and shifting between these different styles of e-cigarettes use. What our case study does show, however, is the fluidity in the ways in which the young people can engage with e-cigarettes over a relatively short period of time. These findings are congruent with the research undertaken by Hair and colleagues [8] in the United States showing the high rate of movement into and out of different patterns of vaping and smoking by adolescents and young adults. Whilst the U.S.-based study by Hair and colleagues was able to show the frequency of transitions in smoking and vaping behaviour amongst young people, they were not able to identify the possible drivers behind such frequent movement. Within our small case study, however, we have been able to show some of the behavioural elements, including the views of peer group members, which might explain why our young people shifted so markedly in their view of vaping over a relatively short period of time.

The young people we interviewed presented vaping as a context driven social activity. At T1, it was associated with spectacle, a means of consumption, and a hobby. By T2, most no longer vaped at all and described the activity in rather pejorative terms. For the two regular smokers, vaping had become an activity no longer associated with parties and display, but with private relaxation and more functionally as a means to ameliorate the harms of tobacco. The interviews at T1 suggested that vaping could be seen as an accepted and integrated part of their socialising with each other. By T2, the case study group had substantially changed their use of, and attitudes towards, vaping. What appeared to be a socially accepted activity at T1 no longer looked to be the case by T2.

These results show just how mutable perceptions and engagement with e-cigarettes can be amongst teens and how much these elements can change over a relatively short period of time.

## 5. Conclusions

This case study has implications with regard to the methods that are used to elicit information on the nature and extent of adolescent use of e-cigarettes. Our research suggests that there is merit in combining questions that are designed to obtain information on the frequency of e-cigarettes use with questions of a more qualitative kind oriented towards eliciting young peoples’ views of their vaping and smoking. Whilst adolescence may not be a period that is unique in the extent to which individuals may be influenced by the reactions and perceptions of other people, our examination of this case study has shown how negative perceptions of vaping came to impact upon the adolescents’ prior positive view of vaping with the result that the teenagers we were interviewing were much more reticent at time two to vape in public settings. The experience of these teens shows the close alignment that can occur between public perceptions of vaping and vaping frequency. The second implication of our research has to do with the marked shift in these adolescents’ perception of vaping over a relatively short period of time. On the basis of these results, there is merit in ensuring that both quantitative and qualitative studies designed to elicit information on the frequency of vaping include, within their design, some element of repeat measures. Repeated measurement is a common element of quantitative surveys but much less common in qualitative research.

On the basis of our case study, vaping needs to be understood as much as a social activity as a means of nicotine delivery. Just as young people can move into using e-cigarettes and other devices, so too can they move out of their use. Issues of fashion, theatricality, risk (of having equipment stolen), poor functionality (of equipment breaking down), reduced adverse health impact (breathing more easily under exertion), the reaction of other people, price, and availability may all impact upon whether e-cigarette use becomes an embedded or a temporary aspect of these young peoples’ social worlds. Our study underscores the importance of differentiating between long-term, frequent, consistent use and more episodic, experimental, and infrequent use by young people and for undertaking measurement of actual e-cigarette use at multiple time points in both quantitative and qualitative studies.

Efforts aimed at addressing the proliferation in e-cigarette use on the part of young people clearly need to focus upon young peoples’ access to these devices. However, on the basis of our results, it is also important to recognise that young peoples’ own peer groups are likely to be an important determinant with regard to both increasing the likelihood of these devices being used and an important tool in reducing their use. Health education interventions aimed at reducing e-cigarette use by young people may well need to better understand how to engage with peer groupings as co-contributors to efforts at reducing e-cigarette use by non-smoking teens in particular, rather than simply exhorting those teens not to use e-cigarettes.

## Figures and Tables

**Table 1:** Reported change in vaping and smoking at six months (Time = T1/T2).

Name	Age		Currently Smoking		Smokes at Parties		Vapes at Least Weekly	
	T1	T2	T1	T2	T1	T2	T1	T2
Daniel	16	16	x	✓	✓	✓	✓	✓
Thomas	16	17	✓	✓	✓	✓	✓	✓
Gayle	16	16	x	x	x	x	x	x
Lyra	16	17	x	x	✓	✓	✓	x
Martyn	16	-	✓	-	✓	-	✓	-
Gavin	16	16	x	x	x	✓	✓	x
Philip	16	17	x	x	x	✓	x	x
Josh	16	16	x	x	x	x	✓	x
